# Circulating NK cells and their subsets in Behçet's disease

**DOI:** 10.1111/cei.12939

**Published:** 2017-03-13

**Authors:** M. S. Hasan, P. L. Ryan, L. A. Bergmeier, F. Fortune

**Affiliations:** ^1^ Centre for Clinical and Diagnostic Oral SciencesInstitute of Dentistry, Barts and The London School of Medicine and Dentistry, Queen Mary University of LondonLondonUK; ^2^ Centre for Adult Oral HealthInstitute of Dentistry, Barts and The London School of Medicine and Dentistry, Queen Mary University of LondonLondonUK

**Keywords:** Behçet's disease, immunopathology, immunotherapies and innate immunity, NK cells

## Abstract

Behçet's disease (BD) is an autoinflammatory, chronic relapsing/remitting disease of unknown aetiology with both innate and acquired immune cells implicated in disease pathogenesis. Peripheral blood natural killer (NK) cells and their CD56^Dim^/CD56^Bright^ subsets were surface phenotyped using CD27 and CD16 surface markers in 60 BD patients compared to 60 healthy controls (HCs). Functional potential was assessed by production of interferon (IFN)‐γ, granzyme B, perforin and the expression of degranulation marker CD107a. The effects of disease activity (BD^Active^ *versus* BD^Quiet^) and BD medication on NK cells were also investigated. Peripheral blood NK cells (*P* < 0·0001) and their constituent CD56^Dim^ (*P* < 0·0001) and CD56^Bright^ (*P* = 0·0015) subsets were depleted significantly in BD patients compared to HCs, and especially in those with active disease (BD^Active^) (*P* < 0·0001). BD patients taking azathioprine also had significantly depleted NK cells compared to HCs (*P* < 0·0001). A stepwise multivariate linear regression model confirmed BD activity and azathioprine therapy as significant independent predictor variables of peripheral blood NK percentage (*P* < 0·001). In general, CD56^Dim^ cells produced more perforin (*P* < 0·0001) and granzyme B (*P* < 0·01) expressed higher CD16 levels (*P* < 0·0001) compared to CD56^Bright^ cells, confirming their increased cytotoxic potential with overall higher NK cell CD107a expression in BD compared to HCs (*P* < 0·01). Interestingly, IFN‐γ production and CD27 expression were not significantly different between CD56^Dim^/CD56^Bright^ subsets. In conclusion, both BD activity and azathioprine therapy have significant independent depletive effects on the peripheral blood NK cell compartment.

## Introduction

Behçet's disease (BD) is a chronic relapsing/remitting multi‐system disease of poorly understood aetiology, characterized primarily by orogenital ulceration, but also affecting other body systems, including the eye (uveitis), joints (arthritis) and skin 1, 2. BD can be life‐threatening in severe forms where the disease progresses to involve the large blood vessels, central nervous system or the gastrointestinal tract. The disease is increased approximately sixfold in patients with human leucocyte antigen (HLA)‐B51/B5 genetic polymorphisms 3 and is regarded widely as an autoinflammatory condition, although autoimmune responses to certain specific antigens have been described in the disease 4, 5. Viral infection with Epstein–Barr virus (EBV) and herpes simplex virus (HSV) were also thought to be important in both the initiation and in triggering acute exacerbations of affected systems in BD 6, 7 and natural killer (NK) cells are important in controlling viral infections.

Loss of normal immune regulation is thought to play a key role in BD pathology, with neutrophil activation and recruitment to the site of inflammatory lesions thought to be central in the disease process 8, 9. Additionally, T helper type 1 (Th1)/Th17 cytokine polarization of CD4^+^ T cells is a consistent finding in disease lesions and in peripheral blood where increased interferon (IFN)‐γ, tumour necrosis factor (TNF)‐α, interleukin (IL)‐8 and IL‐17 levels have been correlated with BD activity 10. Conversely, a reduction in the regulatory T cells (T_regs_) and the cytokine IL‐10 has also been described in the disease 11, 12. While BD lesions are predominated by neutrophils and CD4^+^ T cells, innate lymphoid cells including γδ T cells and conventional NK cells are also found in BD lesions, and may play a significant role in driving the CD4^+^ Th1 response characteristic of BD lesions 13, 14.

NK cells are CD3^–^ granular cytotoxic lymphocytes included in the type 1 group of innate lymphoid cells with the ability to mount cytotoxic major histocompatibility complex (MHC)‐unrestricted responses against pathogens without prior antigen priming 15, 16, 17. They have been characterized conventionally using CD56 [neural cell adhesion molecule (NCAM)], dividing them into the predominant CD56^Dim^ subset (approximately 90% in peripheral blood) and the minority CD56^Bright^ subset 18. These subsets are both phenotypically and functionally distinct, with the more cytotoxic CD56^Dim^ NK subset displaying increased potential to produce perforin and granzyme B 19, 20. This is in contrast to the CD56^Bright^ NK subset, which is less cytotoxic and is more immunoregulatory in function, with a propensity to produce significantly more IFN‐γ and TNF‐α 21. Two further surface markers that have been used commonly to characterize NK cells and their subsets are the low‐affinity Fc‐receptor, CD16 and the TNF‐receptor co‐stimulatory surface molecule CD27 22, 23. In general, CD56^Dim^ NK cells have higher CD16 expression reflective of their more cytotoxic function, with CD27 expression found mainly on the immunoregulatory, cytokine‐producing CD56^Bright^ subset 23, 24, 25. Peripheral blood circulatory NK cells contain high concentrations of preformed cytotoxic granules (e.g. granzyme B and perforin) in their cytoplasm, which can be used to kill target cells on degranulation into the immunological synapse 26. The marker CD107a (lysosome‐associated membrane protein) lines the internal surface of cytotoxic granules and is externalized to the cell surface upon degranulation, where lysosomes fuse with the cell surface in cytotoxic lymphocytes 27. CD107a expression correlates well with IFN‐γ and TNF‐α cytokine secretion as well as NK cell‐mediated lysis of target cells, and is considered as a more sensitive marker than intracellular cytokine assays or the chromium release assay 28, 29.

NK cell numbers and activity have been reported previously in BD with respect to disease activity and also to the medication used to treat BD patients, e.g. azathioprine, cyclosporin and corticosteroids 30. The numbers and cytotoxic capacity of NK cells reported in BD lesions appear to be variable 14, 31, 32, 33, 34, 35, 36, with uncertainty in the literature due to the use of different markers to phenotype NK cells and their subsets 22, 23, 37. Several studies have found increased NK cell numbers in the peripheral blood of BD patients 14, 32, especially during the active phases of disease 38, while other studies have revealed that BD and matched healthy controls had similar NK cell numbers in peripheral blood 31, 35, 39. However, there is no previous study, to our knowledge, reporting on the proportion and functional potential of NK subsets CD56^Dim^ and CD56^Bright^ in BD. Another recent study in uveitis‐affected BD patients demonstrates that NK cell cytokine production favours Th1/NK1 cytokine production (TNF‐α, IFN‐γ and IL‐2) during periods of activity, with a relative switch towards Th2/NK2 cytokines (IL‐4 and IL‐10) during remission 40.

Therefore, this study investigated peripheral blood NK cells and their CD56^Dim^ and CD56^Bright^ subsets in a large cohort of BD patients compared to healthy controls. Subsets were characterized further by surface expression of CD27 and CD16. Functional potential was assessed by production of IFN‐γ, perforin and granzyme B, with degranulation potential assessed using CD107a. Finally, the effect of various systemic BD medication on the frequency of NK cells was also investigated.

## Materials and methods

### Patients and healthy control cohorts

Peripheral blood was obtained from a BD patient cohort (*n* = 60; 20 males, 40 females; median age = 42 years; range = 18–74 years) and compared to healthy controls (HCs) (*n* = 60; 29 males, 31 females; median age = 33 years, range = 21–69 years). All the patients fulfilled the International Study Group for Behçet's disease criteria (ISG 1990) 41. At the time of peripheral blood sampling, the patients had various degrees of clinical disease activity. For the purposes of this study, BD activity was determined by the presence (BD^Active^, *n* = 44) or absence (BD^Quiet^, *n* = 16) of any single clinically active system, including: oral aphthous or genital ulceration, skin lesions, ocular, vascular, rheumatological, gastrointestinal or neurological involvement on the day of sampling. In addition, the patient's current systemic BD medication was recorded (Table 1). The patient cohort was recruited from those attending the Behçet's Centre of Excellence at The Royal London Hospital (Barts Health NHS Trust). Peripheral blood samples and associated clinical information was collected with informed consent from BD patients and healthy controls. The study was approved by the East London and the City Local Research Ethics Committee in full compliance with the Helsinki Declaration (REC References: P/03/122 and 13/LO/0548).

**Table 1:** Characteristics of the Behçet's disease (BD) patient cohort (BD^Active^ and BD^Quiet^) and healthy controls together with current systemic medication

	HCs (*n* = 60)	BD^Quiet^ (*n* = 16)	BD^Active^ (*n* = 44)	BD^Total^ (*n* = 60)
Median age, years (range)	33 (21–69)	42·5 (26–74)	40 (18–74)	42 (18–74)
Gender (M : F)	29 M, 31 F	6 M, 10 F	14 M, 30 F	20 M, 40 F
No. of systems with BD activity	0	0	1 system (*n* = 16)	
			2 systems (*n* = 6)	
			3 systems (*n* = 15)	
			4 systems *n* = 5)	
			5 systems (*n* = 1)	
			6 systems (*n* = 1)	
BD medication (total no. of patients on each BD‐associated systemic therapy)	n.a	Aza = 5	Aza = 14	Aza = 19
		Col = 5	Col = 9	Col = 14
		Pred = 4	Pred = 12	Pred = 16
		MMF = 3	MMF = 3	MMF = 6
		*Inf = 1	Inf = 0	Inf = 1
		*Met = 0	Met = 1	Met = 1
		No BD meds = 2	No BD meds = 16	No BD meds = 18
BD medication combinations	n.a.	Aza only = 2	Aza only = 6	Aza only = 8
		Col only = 4	Col only = 5	Col only = 9
		MMF only = 2	MMF only = 1	MMF only = 3
		Pred only = 1	Pred only = 4	Pred only = 5
		Inf only = 1	Inf only = 0	Inf only = 1
		Aza+Col = 1	Aza+Col = 3	Aza+Col = 4
		Aza+Pred = 2	Aza+Pred = 4	Aza+Pred = 6
		MMF+Pred = 1	MMF+Pred = 2	MMF+Pred = 3
		Pred+Met = 0	Pred+Met = 1	Pred+Met = 1
		Aza+Pred+Col = 0	Aza+Pred+Col = 1	Aza+Pred+Col = 1
		No BD meds = 2	No BD meds = 16	No BD meds = 18

*Patients on infliximab (*n* = 1) and methotrexate (*n* = 1) were excluded in evaluation of effect on natural killer (NK) cells due to only single individuals in each of these subgroups. Aza = azathioprine; BD = Behçet's disease; col = colchicine; MMF = mycophenolate mofetil; pred = prednisolone; inf = infliximab; met =  methotrexate; no BD meds: = no systemic BD‐associated medication.

### Isolation of peripheral blood mononuclear cells

Peripheral blood mononuclear cells (PBMCs) were isolated by Ficoll‐Paque (GE Healthcare, Amersham, UK) density gradient centrifugation. Peripheral blood was layered onto Ficoll‐Paque and centrifuged at 400 ***g*** (brake‐off) for 35 min at 20°C. The interface containing mononuclear cells was collected and washed in complete media (RPMI‐1640; Lonza, Slough, UK) containing 10% fetal bovine serum (FBS), 2 mM L‐glutamine, penicillin (100 IU/ml) (Gibco, Life Technologies, Paisley UK) and streptomycin (100 µg/ml) (Sigma‐Aldrich, Poole, UK) prior to experimental processing and analysis

### Flow cytometry

Fluorochrome‐conjugated antibodies specific for the following cell surface and intracellular molecules were used: CD3 (HIT3a), CD16 (3G8), CD27 (O323), CD28 (CD28.2), CD56 (HCD56), CD107a (H4A3), granzyme B (GB11), IFN‐γ (4S.B3), perforin (dG9) and Vδ2 (B6) (antibody reagents were purchased from either Becton, Dickinson and Company, Oxford, UK, eBioscience, Lutterworth UK or Biolegend, London, UK). PBMCs were incubated on ice with fluorochrome‐conjugated antibodies diluted in fluorescence activated cell sorter (FACS) buffer [phosphate‐buffered saline (PBS) supplemented with 2% FBS and 5 mM ethylenediamine tetraacetic acid (EDTA) (Gibco, Life Technologies) for cell surface staining. Cells were subsequently washed and resuspended in FACS buffer prior to analysis. For intracellular cytokine staining, PBMCs were stimulated with 50 ng/ml phorbol 12‐myristate 13‐acetate (PMA) (Sigma, Poole, UK) and 1 μg/ml ionomycin (Sigma) for 5 h at 37°C; 10 μl brefeldin A (eBioscience) and 2 μM monensin (eBioscience) were added during the last 2 h. Cells were stained for cell surface markers, fixed with IC fixation buffer (eBioscience) for 15 min on ice, and subsequently permeabilized and stained with intracellular cytokine‐specific antibodies diluted in permeabilization buffer (eBioscience). Flow cytometry was carried out using a FACS Canto II flow cytometer (BD Biosciences) and data were analysed using FlowJo software (Tree Star, Inc., Ashland, OR, USA). NK cells were defined as CD3^–^Vδ2^–^CD56^+^, subdivided into CD56^Dim^ and CD56^Bright^ subsets and expressed relative to total gated lymphocytes. The lymphocyte population was identified by assessment of size and granularity of cells using light‐scatter properties [forward‐scatter (FSC) *versus* side‐scatter (SSC)] and NK percentage expressed as a proportion of total gated lymphocytes. Gates were set using appropriate isotype/negative controls for each intra‐ and extracellular antibody.

### Degranulation assay

PBMCs were stimulated initially for 5 h in complete media with 50 ng/ml phorbol‐12‐myristate‐13‐acetate (PMA; Sigma) and 1 μg/ml ionomycin (Sigma) in the presence of anti‐CD107a (BioLegend) at 37°C in 5% CO_2_. After 1 h of stimulation, brefeldin (10 μg/ml) and monensin (2 μM) were added and were present for the last 4 h of culture. Cells were vortexed periodically to prevent cell settling. PBMCs were then washed and stained for cell‐surface markers as described previously. Finally, cells were fixed with intracellular (IC) fixation buffer (eBioscience) and permeabilized and stained for detection of intracellular granzyme B, as described above.

### Statistical analysis

The results are expressed as mean values ± standard error of mean (s.e.m.). GraphPad Prism version 6 (GraphPad Software, San Diego, CA, USA) was used for statistical analysis. Differences between group variables were analysed using non‐parametric single and multiple comparison statistical tests where appropriate (Mann–Whitney *U*‐test/Kruskal–Wallis test). *P*‐values of < 0·05 were considered statistically significant. Non‐parametric tests were used, as samples were not demonstrated to be distributed normally (Kolmogorov–Smirnov normality test). IBM spss Statistics for Windows, version 22.0 (Armonk/IBM Corp., New York, NY, USA) was used to calculate individual Pearson's correlation coefficients for each investigated predictor variable (gender, age, BD activity and each specific BD medication) against NK percentage. Subsequently, multivariate linear regression analysis was performed to determine the relative effect of each predictor variable on NK percentage using a stepwise regression model.

## Results

### Peripheral blood NK cells are depleted significantly in BD patients and correlates with disease activity

**Figure 1:**
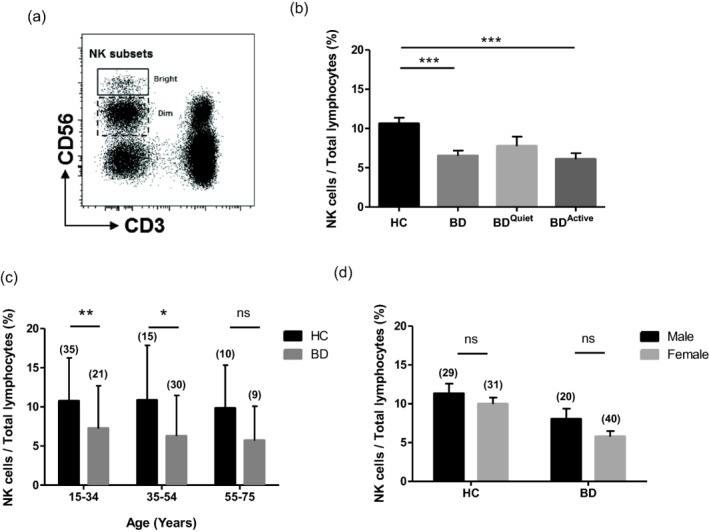
Natural killer (NK) cell peripheral blood percentage decreases independently of age in Behçet's disease (BD)^Active^ patients. (a) NK cells are defined using flow cytometry as CD56^(+)^ CD3^(–)^, with two main subsets characterized by intensity of CD56 expression CD56^Bright^ and CD56^Dim^ (dot‐plot shown from a single healthy control sample to demonstrate population gating). (b) NK cells CD56^+^CD3^–^ are expressed as a proportion of total gated lymphocytes in healthy controls (HC) (*n* = 60) compared to BD patients with no clinical signs of activity BD^Quiet^ (*n* = 16) and those with active systems BD^Active^ patients (*n* = 44) (total BD; *n* = 60). (c) NK/gated lymphocyte (%) is compared in both total BD and HC patients across age ranges 15–34, 35–54 and 55–75 years. CD56^Bright^ NK cells expressed as a proportion of total NK cells is compared between HC and BD patients. (d) Gender differences in NK/gated lymphocyte (%) is compared between HC and BD patients. *P < 0·05; **P < 0·01; ***P < 0·001; n.s. = not significant. Numbers in parentheses above each bar indicate number of individuals in each comparison group.

In accordance with previous literature 18, human natural killer (NK) cells defined in this study as CD3^–^Vδ2^–^CD56^+^ lymphocytes could be separated broadly into CD56^Dim^ and CD56^Bright^ subsets in all Behçet's disease patients (BD) and healthy controls (HC) (Fig. 1a).

NK cells expressed as a percentage of total gated lymphocytes in the peripheral blood of all 60 BD patients (6·53 ± 0·66%) were found to be reduced significantly compared to healthy controls (10·62 ± 0·75%) (*P* < 0·0001). Furthermore, when the total BD cohort was subdivided based on current disease activity, the NK percentage was reduced significantly only in BD patients with active disease (BD^Active^) (6·08 ± 0·78) (*P* < 0·0001), but just failed to reach significance in those without current signs or symptoms (BD^Quiet^) (7·76 ± 1·21) (*P* = 0·0552) (Fig. 1b).

When the HC and BD cohorts were subdivided by age, NK depletion was seen in all three age range groups when BD patients were compared to HCs, reaching statistical significance in the 15–34‐year‐old (*P* = 0·0071) and 35–54‐year‐old subgroups (*P* = 0·0166), but just failing to show significance in the 55–75‐year‐old cohort (*P* = 0·0535). Interestingly, ageing did not affect NK cell percentage significantly, with no significant changes when the three age ranges were compared in HC or BD patients (*P* > 0·05). This suggests that BD‐associated NK depletion appears to be age‐independent (Fig. 1c). Additionally, there were no significant gender differences in NK percentage within HC or BD cohorts or when considered together as a single group (*P* > 0·05) (Fig. 1d).

### CD56^Dim^ and CD56^Bright^ NK cells are decreased significantly in the peripheral blood of BD patients with overall proportional increase in the CD56^Bright^ subset

**Figure 2:**
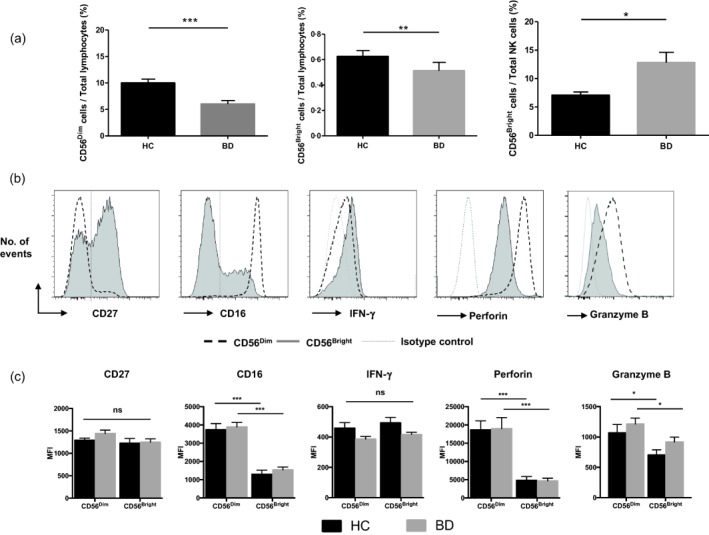
CD56^Dim^ and CD56^Bright^ natural killer (NK) subsets are phenotypically and functionally distinct. (a) Difference in percentage of CD56^Dim^ (far left) and CD56^Bright^ subsets (middle) as a percentage of gated lymphocytes with change in CD56^Bright^ subset expressed as a percentage of total NK cells (far right). (b) Flow cytometry histogram plots showing intensity of surface marker expression of CD27 and CD16 and intracellular cytokine staining for interferon (IFN)‐γ, perforin and granzyme B following 5‐h phorbol myristate actate (PMA)/ionomycin stimulation (representative plots from healthy control sample are shown). (c) Summary median fluorescent intensities (MFI) of gated positive events for CD27, CD16, IFN‐γ, perforin and granzyme B showing relative differences in expression intensities of markers on each of NK subset (CD56^Dim^ and CD56^Bright^) in healthy controls (HC) and in Behçet's disease (BD) patients. *P < 0·05; **P < 0·01; ***P < 0·001; n.s. = not significant.

BD patients had significantly depleted CD56^Dim^ (HC = 9·99 ± 0·74% *versus* BD = 6·02 ± 0·65%) (*P* < 0·0001) and CD56^Bright^ (HC = 0·62 ± 0·05% *versus* BD = 0·51 ± 0·06%) (*P* = 0·0015) NK subsets compared to healthy controls relative to total lymphocytes. Overall, this led to a net increase in the proportion of the CD56^Bright^ subset relative to total NK cells in BD (12·80 ± 1·82%) compared to HC (7·05 ± 0·59%) *(P* = 0·0342) (Fig. 2a). Therefore, BD appears to be associated with a shift in NK subsets with a relative increase in the CD56^Bright^ compared to CD56^Dim^ cells on the background of overall peripheral blood NK depletion.

### CD56^Dim^ and CD56^Bright^ subsets are phenotypically and functionally distinct in both HCs and BD

The expression of surface markers CD27 and CD16 as well as the ability of NK subsets to produce IFN‐γ, perforin and granzyme B and (as measured by median fluorescent intensities of gated positive events) were compared in NK subsets. CD56^Dim^ NK cells were found to express 2·5–2·8 times more surface CD16 (*P* < 0·0001) and produce significantly higher levels of perforin (3·8–4 times) (*P* < 0·0001) and granzyme B (1·3–1·5 times) (*P* < 0·05) compared to CD56^Bright^ cells. By contrast, the levels of CD27 surface expression and production potential of IFN‐γ on a per cell basis was not found to be different between subsets (Fig. 2b, c). These same differences were maintained in CD56^Dim^ and CD56^Bright^ subsets from both HCs and BD patients, with no significant differences in the subsets between HCs and BD on a per cell basis.

### Increased percentage of IFN‐γ producing CD56^Bright^ cells in BD

**Figure 3:**
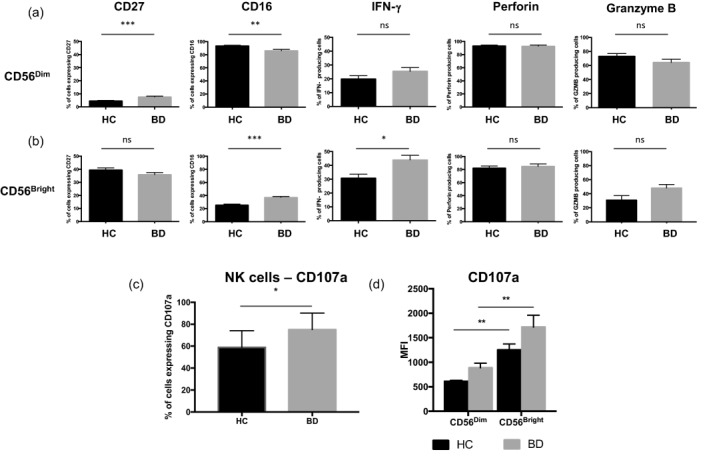
Comparison of percentage expression of CD27, CD16 and interferon (IFN)‐γ between natural killer (NK)^Dim^ and NK^Bright^ subsets. Percentage expression of surface markers CD27 and CD16 alongside intracellular staining for the cytokine IFN‐γ and cytotoxic proteins perforin and granzyme B following 5‐h phorbol myristate acetate (PMA)/ionomycin stimulation for (a) CD56^Dim^ and (b) CD56^Bright^ NK subsets. (c) Percentage CD107a expression is shown on total NK cells in heathy control (HC) individuals (*n* = 6) compared to Behçet's disease (BD) patients (*n* = 14) following 5‐h PMA/ionomycin activation (left panel). Summary median fluorescent intensities (MFI) of gated positive events are shown for CD107a showing relative differences in expression intensities of markers on each of NK subset (CD56^Dim^ and CD56^Bright^) in HC and in BD patients. *P < 0·05; **P < 0·01; ***P < 0·001; n.s. = not significant.

When intracellular expression of IFN‐γ was analysed, the percentage of IFN‐γ producing CD56^Dim^ and CD56^Bright^ NK cells was higher in BD patients compared to HCs, but this only reached statistical significance in the CD56^Bright^ subset (BD 43·92 *versus* HCs 30·72%) (*P* = 0·0255) (Fig. 3a, b). By contrast, no differences in the percentage of perforin‐ and granzyme B‐producing cells was observed between HCs and BD patients in either subset, with the majority of NK cells demonstrating a robust ability to produce these cytotoxic proteins. Furthermore, no significant difference in IFN‐γ, perforin or granzyme B was observed with respect to BD disease activity (BD^quiet^ compared to BD^Active^) (data not shown).

### NK subsets show opposing trends in percentage of CD56^Dim^ and CD56^Bright^ cells expressing CD27 and CD16

The percentage of CD56^Bright^ NK cells expressing CD16 was significantly higher in BD patients (37·14 ± 1·47%) when compared with HC (25·40 ± 1·45%) (*P* < 0·0001) (Fig. 3b). This was in direct contrast to the CD56^Dim^ subset which, although this had a higher and predominant expression of surface CD16, showed a significant decrease in surface expression in BD (86·10 ± 2·09%) compared to HCs (93·54 ± 0·87%) (*P* = 0·0032) (Fig. 3a). However, the opposite pattern of change was observed in CD27 expression where the percentage of CD56^Dim^ subset expressing CD27 was increased in BD (7·60 ± 0·64%) compared to HCs (4·58 ± 0·33%) (Fig. 3a). In contrast, the CD56^Bright^ subset showed the opposite trend (BD 35·86 *versus* HCs 39·50%), which failed to reach statistical significance *(P* = 0·07) (Fig. 3b).

### Increased percentage of NK cells with degranulation potential in BD patients compared to HC individuals

Total NK cells exhibited higher overall percentage expression of the degranulation marker CD107a following activation in BD compared to HC individuals (*P* = 0·026) (Fig. 3c), with no significant further increase in BD^Active^ compared to BD^Quiet^ patients (*P* > 0·05). However, when NK subsets were compared on a per cell basis, CD56^Bright^ subset expressed significantly higher levels of CD107a compared to the CD56^Dim^ subset within both the HC and BD groups (*P* < 0·01). Overall, the CD107a expression on a per cell basis was similar in both NK subsets between HC and BD patients (*P* > 0·05) (Fig. 3d).

### NK depletion is greatest in BD patients on current azathioprine therapy

**Figure 4:**
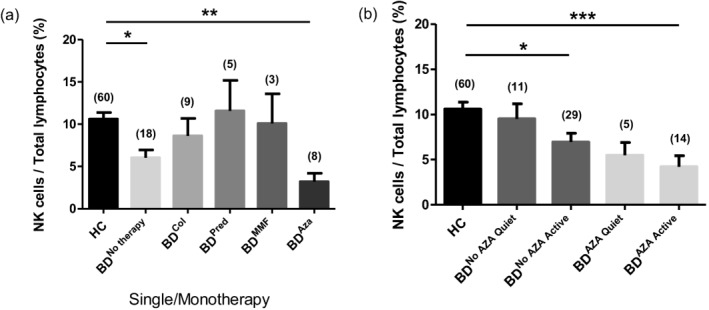
Natural killer (NK) cell peripheral blood percentage is reduced significantly in patients under azathioprine therapy. (a) NK cells expressed as a percentage of total gated lymphocytes was compared in patients on different forms of Behçet's disease (BD) medication; colchicine (BD^Col^), prednisolone (BD^Pred^), mycophenolate mofetil (BD^MMF^) and azathioprine (BD^Aza^) and compared to healthy controls (HC) as well as patients not currently taking BD‐associated medication (BD^No therapy^). (b) NK cell percentage expressed as a percentage of total lymphocytes in BD patients subgrouped according to disease activity (BD^Quiet/Active^) and whether currently taking azathioprine or not (BD^Aza^ or BD^Non‐Aza^). *P < 0·05; **P < 0·001; ***P < 0·001. Aza = azathioprine; Col = colchicine; MMF = mycophenolate mofetil; Pred = prednisolone; Inf = infliximab; Met = methotrexate; no BD meds = no systemic BD‐associated medication. Patients on infliximab (*n* = 1) and methotrexate (*n* = 1) were excluded in evaluation of effect on NK cells due to only single individuals in each of these subgroups. Numbers in parentheses above each bar indicate number of individuals in each comparison group.

In order to determine whether NK cell depletion was due to disease activity or as a result of specific immunomodulatory medication, the BD cohort was subdivided according to current systemic BD drug therapy (azathioprine, colchicine, prednisolone and mycophenolate mofetil). The effect of these medications (when used as single/monotherapy) on NK percentage was compared with both HCs and non‐medicated BD patients (Fig. 4a).

NK cells/total lymphocytes (%) were reduced significantly only in azathioprine‐treated BD patients compared to HCs (*P* < 0·01) but, interestingly, not for BD patients on other forms of therapy (BD^Col^, BD^Pred^, BD^MMF^) (Fig. 4a). Importantly, BD patients not currently receiving active drug therapy (BD^No therapy^) also demonstrated reduced percentages of NK cells in comparison with HCs (*P* < 0·05) (Fig. 4a).

Furthermore, when BD patients were subgrouped according to both disease activity (BD^Quiet^/BD^Active^) and whether or not they were taking azathioprine (BD^Aza^/BD^Non‐Aza^), it was apparent that the two groups with the lowest NK percentages were both currently taking azathioprine (BD^Aza/Quiet^ and BD^Aza/Active^) (Fig. 4b). However, BD^Non‐AZA/Active^ patients also showed significantly depleted percentages of NK cells compared to HCs (Fig. 4b).

We further investigated the relative importance of the following predictor variables (gender, age, BD activity and BD medication) in relation to peripheral blood NK percentage by initially examining the Pearson correlation coefficients of each predictor variable against NK percentage. This revealed BD activity, azathioprine therapy and gender to be correlated significantly with NK percentage (*P* < 0·05) (Table 2). However, when stepwise multivariate linear regression was used to generate the regression model, only BD activity and azathioprine therapy were determined as having significant depletive effects on the NK cell percentage from the variables analysed (*P* < 0·05) (Table 2). It appears that both the systemic BD activity and azathioprine medication seem to have independent depletive effects on peripheral blood NK cells. However, although statistically significant, the regression model incorporating these two variables together only explained 15·2% of the variation on NK percentage (*R* ^2^ = 0·152, *P* < 0·001***).

**Table 2:** Bivariate and multivariate linear regression investigating the relationship between various predictor variables and natural killer (NK) cell percentage

	Correlation		Multiple linear regression		
Variable	Pearson	*P*‐value	β‐coefficient	95% CI	*P*‐value
Gender	−0·203	0·013*			
Age	−0·070	0·225			
BD activity	−0·352	<0·001***	−3·070	−5·275 to −0·864	**0·007
Azathioprine	−0·315	<0·001***	−2·905	−5·807 to −0·003	*0·049
Prednisolone	−0·115	0·106			
Colchicine	−0·127	0·084			
MMF	−0·085	0·178			

Individual Pearson's correlation coefficient statistics with associated *P*‐values for each independent predictor variable of NK cell percentage alongside stepwise multiple linear regression model (*R* ^2^ = 0·152, *F*_(2, 117)_ = 10·511, *P* = 0·000063 (*P* < 0·001***). BD = Behçet's disease; CI = confidence interval; MMF = mycophenolate mofetil.

## Discussion

This current cross‐sectional experimental study represents the largest study to date investigating NK cells, their subsets and function in relation to BD systemic clinical activity and common BD medication. While a number of previous studies have investigated NK cells in BD patients 14, 32, the literature is somewhat contradictory, due probably to the heterogeneity of the BD patient cohort in terms of age, ethnicity and varied clinical presentation overlaid with an often‐complex drug management regimen. This unclear picture is reflected in the findings of previous studies that both support 35 and contradict 14, 32 NK cell peripheral blood depletion and activity in BD.

In this study, both CD56^Dim^ and CD56^Bright^ NK cells were found to be depleted within the peripheral blood compartment of BD patients compared to healthy controls. Indeed, NK cell depletion was not only associated with patients diagnosed with BD but, interestingly, was more marked in patients with current disease activity (BD^Active^) compared to those diagnosed previously with BD but without current active disease (BD^Quiet^). Overall, there was depletion of both CD56^Dim^ and CD56^Bright^ NK subsets, with a proportionately greater loss of the CD56^Dim^ subset resulting in an overall increase in the proportion of CD56^Bright^ cells relative to the total NK population.

NK cell depletion also appeared to be most marked in BD patients taking azathioprine compared to those on non‐azathioprine BD medication. This finding is supported by a number of other studies that observed NK depletion and reduced NK activity following azathioprine therapy for rheumatoid arthritis 42, inflammatory bowel disease 43 and in systemic lupus erythematosus 44. Therefore, it is apparent from this study that both disease activity and azathioprine therapy seem to have an independent depletive effect on NK cells, which was confirmed by multivariate analysis. Azathioprine, a purine analogue, is thought to work by blocking the *de‐novo* pathway of purine synthesis which contributes to its relative specificity to lymphocytes 45. It has also been shown that azathioprine inhibits proliferation in resting or newly activated cells but not pre‐activated cells through an increase in apoptosis 46. Therefore, if a BD patient has experienced an event that triggers relapse, azathioprine would then eliminate newly activated cells. The cells that were already activated will not be affected by azathioprine and might be leaving the circulation for trafficking into the tissues. While azathioprine inhibits cell proliferation, colchicine acts primarily by inhibiting microtubule polymerization, which results in decreased cytokine secretion and migration 47. Interestingly, reduced NK activity has also been observed in mycophenolate mofetil (MMF) therapy 48 as well as in patients on prednisolone therapy in previous studies, but was not observed within this cohort 49. Prednisolone, a synthetic glucocorticoid, interferes primarily with the cellular components of the microcirculation following an inflammatory response. This results in decreased vasodilatation, vascular permeability and suppression of leucocyte emigration 50. Similarly, MMF is thought to supress recruitment to inflammatory sites by inhibiting glycosylation of adhesion molecules, which in this case might maintain the circulating pool of NK cells rather than allow them to migrate to inflamed tissues 51.

NK cell peripheral blood depletion in BD pathology therefore suggests different possibilities in terms of mechanistic explanation. One possibility is that BD directly causes NK cell depletion due to net trafficking from peripheral blood into disease active tissue sites, and that BD activity might correlate with an overall net loss of NK cells from the peripheral blood compartment. This perhaps mirrors other immunological NK‐associated diseases such as psoriasis 52, rheumatoid arthritis (RA) 53 and type 1 diabetes mellitus (T1DM) 54, where this has similarly been observed. Indeed, there is further supporting evidence for NK tissue trafficking in active BD, with NK cells present in histological BD samples in eye lesions (uveitis) 55, oral aphthous ulcers and erythema nodosum 14. An alternative possibility is that NK cells are depleted in both peripheral blood as well as in tissue sites as a direct consequence of BD pathology, conceivably leaving individuals susceptible to chronic viral infections, a commonly hypothesized environmental trigger of BD. We have confirmed that depletion of NK cells, both in their number and function, occurs in the BD patients. This may be a result of the underlying immune‐pathogenic process and/or consequences of azathioprine therapy in BD.

As mentioned previously, viral infectious agents such as herpes simplex virus (HSV) and Epstein–Barr virus (EBV) have been suggested as possible environmental triggers of BD 6, 7. Therefore, it follows that the protective response from anti‐viral immune cells such as NK cells may play a key role in regulating BD activity. Indeed, the pathological and immunological consequences of NK cell depletion has been studied in both animal and human studies, highlighting the key role NK cells play in anti‐viral immunity 56, 57. While NK cells have an obvious role in the direct lysis of virally infected cells, they also indirectly play a key role in immunoregulating and dampening down anti‐viral CD4^+^ and CD8^+^ T cell responses 58, 59. In addition, NK cells have also been shown capable of careful regulation of T cell subpopulation specifically controlling Th1/T_reg_ as well as the Th17/T_reg_ balance in one infection model 60. Therefore, it is apparent that NK cells are not only important in direct anti‐viral responses; they are also key regulatory cells in controlling acquired specific immune responses during viral infection and consequently may be key cells in having a role in controlling BD activity.

NK depletion in BD was accompanied by a reduction in the percentage of cells expressing CD16 and an increase in CD27 percentage expression within the predominant CD56^Dim^ NK subset. In contrast, CD16^+^CD56^Bright^ NK cells were increased in BD, suggesting the potential for either direct lysis or antibody‐dependent cell‐mediated cytotoxicity (ADCC) killing by this subset, which has been shown to be an intermediate stage of NK cell differentiation 61. Additionally, the percentage of CD56^Bright^ NK cells with IFN‐γ production potential increased in BD patients, which supports previous evidence indicating a role for NK cells in Th1 cytokine production in BD 13. Indeed, the switch to increased IFN‐γ production is also seen in conventional CD4^+^ T cells in active BD 13, 62, 63. However, on a per cell basis we did not observe significantly increased IFN‐γ production by the CD56^Bright^ subset, as has been observed in other studies 20, 21.

This study also supports the conventional literature that stimulated CD56^Dim^ NK cells produce higher levels of cytotoxic mediators (i.e. perforin and granzyme B) and express significantly more CD16 compared to CD56^Bright^ cells. However, while appearing to have greater ability to produce more intracellular perforin and granzyme B, the degranulation potential (CD107a expression) of this CD56^Dim^ subset was reduced compared to CD56^Bright^ cells. Indeed, this inverse relationship between levels of intracellular cytotoxic protein and CD107a expression have been observed previously when cytotoxic lymphocytes are activated with phorbol myristate acetate (PMA) and ionomycin 64. This observation may simply reflect differences in granule release kinetics during the 5 h of stimulation, inherent differences in granule CD107a receptor density or rate of internalization of the marker. Finally, when NK cells were considered as a single population, the percentage CD107a expression was found to be significantly higher in BD patients compared to HC individuals, suggesting that NK cells are inherently more cytotoxic in BD compared to health. As mentioned previously, CD107a has been shown to be a more sensitive marker of NK activity when compared to intracellular cytokine and chromium release assays, and so may represent the most important functional cytotoxic marker 28.

In summary, this present study suggests that both CD56^Dim^ and CD56^Bright^ subsets are depleted in the peripheral blood of BD patients and that depletion is more marked in patients with active disease as well as in BD patients taking azathioprine. NK peripheral blood depletion in BD patients therefore may reflect an increased homing of these cytotoxic cells to sites of inflammation in active BD initiating and maintaining tissue inflammation through production of Th1 cytokines and cytotoxic mediators.

Further studies are clearly required to better understand the mechanism of NK depletion in BD patients and the relative influence of disease activity and azathioprine medication on NK cells within the peripheral blood as well as in the disease affected tissues.

## Disclosure

The authors have no disclosures nor conflicts of interest to declare.

## Author contributions

M. S. H. and P. L. R. performed the experiments and analysed the data. P. L. R. and M. S. H produced the figures. P. L. R., M. S. H., L. A. B. and F. F. wrote the manuscript.
